# BMP-2 Long-Term Stimulation of Human Pre-Osteoblasts Induces Osteogenic Differentiation and Promotes Transdifferentiation and Bone Remodeling Processes

**DOI:** 10.3390/ijms23063077

**Published:** 2022-03-12

**Authors:** Lena-Christin Ingwersen, Marcus Frank, Hendrik Naujokat, Klaas Loger, Rainer Bader, Anika Jonitz-Heincke

**Affiliations:** 1 Biomechanics and Implant Technology Research Laboratory, Department of Orthopaedics, Rostock University Medical Center, 18057 Rostock, Germany; rainer.bader@med.uni-rostock.de; 2 Medical Biology and Electron Microscopic Center, Rostock University Medical Center, 18057 Rostock, Germany; marcus.frank@med.uni-rostock.de; 3 Department of Life, Light and Matter, University of Rostock, 18059 Rostock, Germany; 4 Department of Oral and Maxillofacial Surgery, University Hospital of Schleswig-Holstein, 24105 Kiel, Germany; hendrik.naujokat@uksh.de (H.N.); klaas.loger@uksh.de (K.L.)

**Keywords:** BMP-2, pre-osteoblasts, osteoblastogenesis, bone regeneration, bone homeostasis, intracellular vacuoles

## Abstract

Bone morphogenic protein (BMP-) 2 plays an important role in the regeneration of bone defects by promoting osteogenic differentiation. However, several animal studies have reported adverse side effects of BMP-2, including osteoclast activation, induction of peroxisome proliferator- activated receptor gamma (PPARG)expression, and inflammation. High BMP-2 concentrations are thought to be responsible for these side effects. For this reason, primary pre-osteoblasts were exposed to lower BMP-2 concentrations (1 and 2 µg/mL). Long-term exposure (up to 28 days) was performed to investigate whether this stimulation protocol may promote osteogenic differentiation without causing the side effects mentioned above. The results showed that BMP-2 treatment for 14 or 28 days resulted in increased osteogenesis, through an increase in runt-related transcription factor 2, osterix, alkaline phosphatase, and integrin-binding sialoprotein expression. However, an increase in tumor necrosis factor alpha and receptor activator of nuclear factor kappa-Β ligand protein levels was observed after BMP-2 exposure, indicating also an increased potential for osteoclast activation by osteoblasts. Additionally, morphological changes like intracellular, filled vacuoles could be detected. Enhanced PPARG and perilipin 1 mRNA transcripts and lipid droplets indicated an induced adipogenic differentiation. Overall, the data demonstrate that long-term BMP-2 exposure promotes not only osteogenic differentiation but also adipogenesis and regulates mediators involved in osteoclast activation in vitro.

## 1. Introduction

In the reconstruction of mandibular defects caused by trauma, infection, tumors, or congenital malformation, the aim is to rehabilitate the defect in an aesthetically and functionally satisfactory manner. According to current standards, autologous bone grafts, e.g., from the fibula, iliac crest, or scapula are used for therapy [1,2,3]. However, a disadvantage of this form of therapy is the partially insufficient fit and the high morbidity caused by bone harvesting. Therefore, the goal of ongoing research is to develop synthetic implants from reconstruction materials that meet the requirements of jaw reconstruction on a biological, physical, and biomechanical level [3,4].

Osteogenic differentiation, in general, is organized in three developmental stages: (i) cell proliferation, (ii) extracellular matrix deposition and matrix maturation, and (iii) matrix mineralization [5]. During these stages, different proteins are expressed. In the early stage, pre-osteoblasts are characterized by active proliferation and expression of collagen, fibronectin, and transforming growth factor—β (TGF-β) receptor 1 [6,7,8]. Later on, proliferation is downregulated and expression rates of collagen type 1 alpha chain (COL1A1) and alkaline phosphatase (ALPL) increase [6,7,8]. In the end, matrix maturation gets completed via the expression of various osteoblastic markers, like osteopontin (OPN), osteocalcin (OC), integrin-binding sialoprotein (IBSP), ALPL, and COL1A1 [6,9]. OPN thereby promotes bone formation and mineralization, whereas IBSP regulates the hydroxyapatite crystal formation [10,11]. During the entire process, the transcription factors runt-related transcription factor 2 (RUNX2) and osterix (OSX) function as master regulators [12,13,14,15,16].

Bone morphogenic protein (BMP-) 2 as a member of the transforming growth factor-beta superfamily is of interest for the biological functionalization of synthetic materials, since BMP-2 plays an important role in bone remodeling and homeostasis in adulthood [17]. BMP-2 forms homo- or heterodimers [18], which then leads to signaling pathway activation in osteoblasts via binding to the corresponding receptors, complexes of BMP type I and BMP type II receptors [19]. BMP-2 acts on the developmental stages of osteoblasts via different signaling pathways, depending on the conformation of BMP receptors [20]. One is the phosphorylation of Smad1, Smad 5, or Smad 8, complex formation with Smad4, and the translocation into the nucleus, which finally leads to the transcription of downstream genes. Furthermore, p38 or c-Jun N-terminal kinases via mitogen-activated protein kinase (MAPK) can be activated [21], and lastly, there is also the possibility that protein kinase A (PKA) gets activated leading to extracellular signal-regulated kinases (ERK) 1/2 MAPK activation [22,23,24].

In addition to the bone-forming properties of BMP-2, several animal studies have reported adverse side effects, including osteoclast activation, induction of peroxisome proliferator-activated receptor gamma (PPARG) expression, and inflammation [25]. Here, high BMP-2 concentrations in a milligram range are thought to be responsible for these side effects in humans [26]. In a rat femoral segmental defect model, total doses of 11.25, 22.5, and 75 µg fused the defect but also led to the formation of adipose tissue [27]. 

The purpose of our present study was to examine the long-term effect of BMP-2 stimulation in human pre-osteoblasts. Our hypothesis is that BMP-2 concentration of 1 µg/mL (in total 200 ng) and 2 µg/mL BMP-2 (in total 400 ng) are high enough to have a positive influence on the osteogenic differentiation potential of human primary pre-osteoblasts in vitro. Different markers on gene expression and protein level were analyzed. At the same time, possible effects of BMP-2 on the induction of osteoclastic processes were considered. For this purpose, osteoprotegerin (OPG) and receptor activator of nuclear factor-kappa B ligand (RANKL), released by pre-osteoblasts, were quantified. Furthermore, the effect on cellular stress level and cell morphology was investigated. Overall, this study aimed to obtain an overview of the long-term effects of BMP-2 in pre-osteoblasts and to investigate whether this stimulation protocol promotes osteogenic differentiation without causing adverse side effects in vitro.

## 2. Results

### 2.1. Effect of BMP-2 on Viability and ALP Activity in Human Pre-Osteoblasts

**Figure 1:**
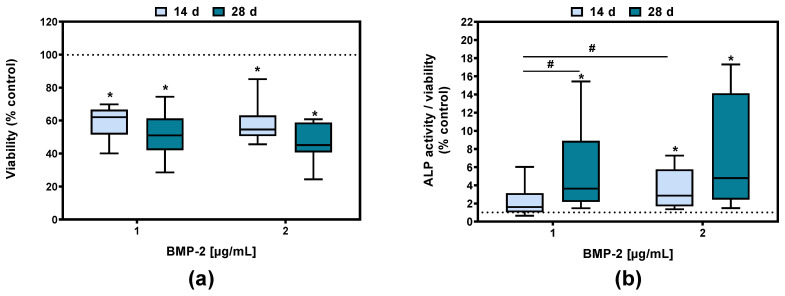
Viability (**a**) and ALP activity related to viability (**b**) of human pre-osteoblasts following BMP-2 exposure to 1 or 2 µg/mL after 14 or 28 days. The data are depicted relative to the control values. Results are depicted as medians, interquartile ranges, minimum, and maximum with * *p* < 0.05 compared to the control and ^#^ *p* < 0.05 compared to different time points or concentrations using two-way ANOVA with Bonferroni’s multiple comparisons test (n ≥ 7).

The viability of human pre-osteoblasts after 14 and 28 days of BMP-2 exposure was significantly decreased [1 µg/mL: *p* = 0.0003 (14 d) and *p* < 0.0001 (28 d); 2 µg/mL: *p* = 0.0002 (14 d) and *p* < 0.0001 (28 d)] compared to the control (indicated as the dotted line). Between the different BMP-2 concentrations no significant effect on metabolic activity was detected (Figure 1a). The quantification of ALP activity after BMP-2 treatment revealed a significant increase after 14 days for 2 µg/mL (*p* = 0.0285) and after 28 days for 1 and 2 µg/mL BMP-2 (*p* = 0.0200 and *p* = 0.0172) compared to unstimulated cells (Figure 1b). A concentration-dependent effect between 1 and 2 µg/mL BMP-2 after 14 days of stimulation could be detected.

### 2.2. Osteoblastic Differentiation and Induction of Bone Remodeling Markers following BMP-2 Exposure

**Figure 2:**
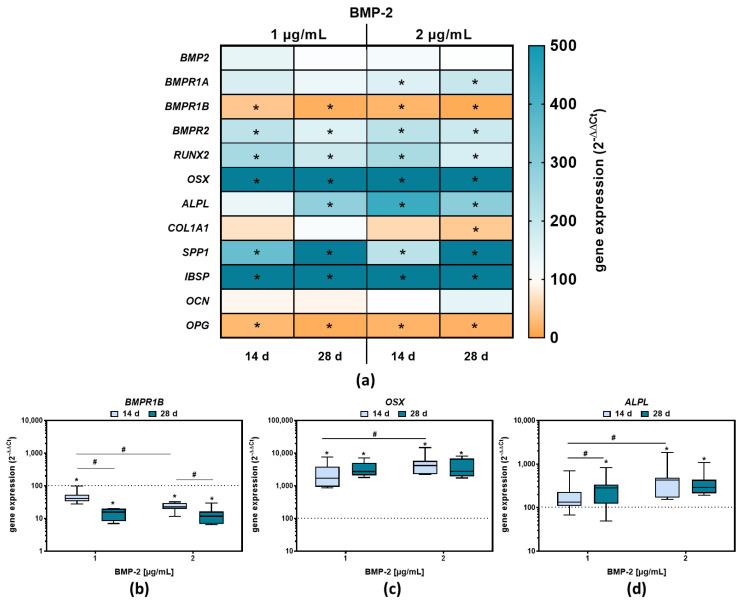
Gene expression results following BMP-2 exposure to 1 or 2 µg/mL after 14 or 28 days. (**a**) Gene expression results depicted as Heatmap. (**b**–**d**) The data are depicted as 2^(−∆∆Ct)^ values with * *p* < 0.05 compared to the control and ^#^ *p* < 0.05 compared to different time points or concentrations using two-way ANOVA with Bonferroni’s multiple comparisons test (n ≥ 7).

Gene expression results of BMP-2 stimulated pre-osteoblasts are depicted in Figure 2a. For visual simplification, a heatmap plot was chosen. Color coding corresponds to the median of at least seven independent osteoblast donors, with a white background corresponding to the unstimulated control (“100%”). If the gene expression rate of defined markers is downregulated compared to the control, they are displayed in orange, with “0” corresponding to no expression. Conversely, up-regulation is highlighted with a blue color code (up to max. “500%”, corresponding to a fivefold increase).

BMP-2 exposure for 14 or 28 days did not increase the expression of *BMP-2* mRNA in pre-osteoblasts compared to the unstimulated control (Figure 2a). The mRNA levels for *BMPR1A* and *BMPR2*, both coding for BMP receptors, were significantly elevated after BMP-2 exposure with 2 µg/mL for 14 or 28 days compared to the control (*BMPR1A*: *p* = 0.0414 (14 d), *p* = 0.025 (28 d); *BMPR2*: *p* = 0.0002 (14 d), *p* = 0.0007 (28 d)). In contrast, the gene expression of *BMPR1B*, also coding for an BMP-2 receptor, was significantly decreased after 14 days of BMP-2 stimulation compared to the control (*p* < 0.0016 (1 µg/mL), *p* < 0.0001 (2 µg/mL) and a concentration-dependent effect was detectable (*p* < 0.0001 (14 d), *p* = 0.0086 (28 d)) (Figure 2a,b). The longer stimulation time of 28 days even enlarged the effect of a decreased *BMPR1B* mRNA expression. Thus, the combination of BMPR1A and BMPR2 appears to be the favored receptor combination in BMP-2 signaling.

The mRNA expression of *RUNX2* and *OSX*, coding for two important osteogenic transcription factors, was significantly increased after BMP-2 exposure with 1 or 2 µg/mL compared to the unstimulated control (*RUNX2* (14 d): *p* < 0.0001 (1 µg/mL), *p* < 0.0001 (2 µg/mL); *OSX* (14 d): *p* < 0.0001 (1 µg/mL), *p* < 0.0001 (2 µg/mL)). This effect was already present after 14 days and could not be further enhanced by a longer exposure time (Figure 2a,c). For *OSX*, a concentration- (*p* = 0.0289) but no time-dependent effect on day 14 was detectable (Figure 2c).

**Figure 3:**
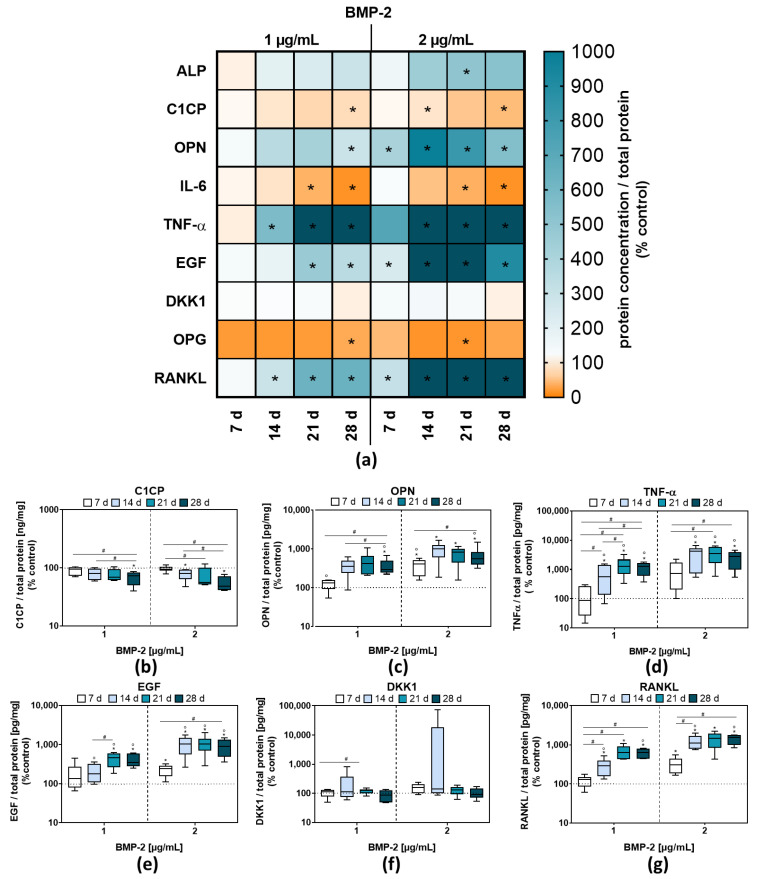
Protein concentrations in the supernatants of BMP-2 stimulated pre-osteoblasts after 7, 14, 21, or 28 days. (**a**) Data of secreted proteins depicted as heatmap. (**b**–**g**) Results of selected proteins secreted by human pre-osteoblasts. All data are depicted in relation to the total protein amount and relative to the control values. Results are shown as medians in the heatmap, * *p* < 0.05 compared to the control and ^#^ *p* < 0.05 compared to different time points and ° *p* < 0.05 compared to different concentrations using two-way ANOVA with Bonferroni’s multiple comparisons test (*n* = 6).

The *ALPL* mRNA expression was significantly increased with 2 µg/mL BMP-2 at both analysis time points (*p* < 0.0001 (14 d), *p* < 0.0001 (28d)) and with 1 µg/mL BMP-2 after 28 days (*p* < 0.0001) compared to the unstimulated control (Figure 2a,d). After 14 days a significant difference in *ALPL* mRNA levels between 1 and 2 µg/mL BMP-2 was detected (*p* = 0.0012). In addition, there was a significant increase of *ALPL* mRNA expression at 1 µg/mL BMP-2 between 14 and 28 days (Figure 2d). Consistent with this, the enzymatic activity of ALP was also increased which was significant for 2 µg/mL BMP-2 after 21 d (*p* = 0.013) (Figure 3a). Unlike the *ALPL* gene expression rates, *COL1A1* gene expression was not significantly influenced by BMP-2 treatment compared to unstimulated cells (Figure 2a). These results were confirmed by protein results of CICP, the C-propeptide of type I procollagen. The protein secretion of CICP was decreased after 28 days following BMP-2 stimulation, either with 1 µg/mL (*p* = 0.0388) or 2 µg/mL (*p* = 0.0098), compared to untreated cells (Figure 3a,b).

To determine whether a long-term BMP-2 stimulation of pre-osteoblasts resulted in the release of pro-inflammatory mediators, protein contents of interleukin (IL-) 6 and tumor necrosis factor alpha (TNF-α) were quantified. For IL-6, a significant reduction in protein secretion after 21 (1 µg/mL: *p* = 0.016; 2 µg/mL: *p* = 0.0028) and 28 days (1 µg/mL: *p* < 0.0001; 2 µg/mL: *p* < 0.0001) following BMP-2 exposure was detectable compared to the control (Figure 3a). In contrast to that, the secretion of TNF-α protein was highly upregulated after 14, 21 and 28 days of BMP-2 exposure (1 µg/mL: *p* = 0.0177 (14 d), *p* = 0.0011 (21 d), *p* = 0.0002 (28 d); 2 µg/mL: *p* = 0.0081 (14 d), *p* = 0.001 (21d), *p* = 0.0006 (28 d)) (Figure 3a,d).

Osteopontin (*SPP1*) mRNA transcripts were significantly increased after BMP-2 treatment compared to the unstimulated control (1 µg/mL: *p* = 0.0446 (14 d), *p* = 0.0002 (28 d); 2 µg/mL: *p* = 0.0399 (14 d), *p* = 0.0004 (28 d) (Figure 2a). The quantification of OPN, the protein encoded by SPP1, showed an increase in protein concentrations after exposure to 2 µg/mL BMP-2 at all-time points (*p* = 0.0139 (7 d), *p* = 0.0182 (14 d), *p* = 0.007 (21 d), *p* = 0.0002 (28 d), whereas at 1 µg/mL BMP-2 stimulation, a significant increase could only be shown after 28 days compared to the control (*p* = 0.0114) (Figure 3a,c).

The *IBSP* gene expression after BMP-2 treatment was strongly increased compared to the unstimulated control regardless of time and concentration (Figure 2a), indicating a continuing osteogenic differentiation, whereas, for the *OCN* gene, coding for osteocalcin, no change in gene expression compared to control cells was observed (Figure 2a). In contrast to that of the mRNA level of *OPG*, a negative regulator of osteoclastogenesis, was reduced at either both time points or both BMP-2 concentrations compared to the control (Figure 2a). The quantification of OPG protein also revealed a reduction in protein contents in the supernatants of BMP-2 stimulated pre-osteoblasts. Consistent with this, the concentration of secreted RANKL, the second osteoclastogenic regulator protein of osteoblasts, was significantly increased after BMP-2 exposure (Figure 3a,g).

Furthermore, Dickkopf-related protein (DKK) 1, an inhibitor of Wnt/β-catenin signaling was analyzed. Activated Wnt signaling through β-catenin-dependent signals induces bone formation through the promotion of osteoblastogenesis and OPG expression. An increase in DKK1 protein concentration would indicate an inhibition of osteogenic differentiation [28], which cannot be observed in our study (Figure 3a,f). Additionally, epidermal growth factor (EGF) is capable of suppressing Wnt/β-catenin-induced osteoblast differentiation [29]. EGF concentration was significantly increased after 1 µg/mL BMP-2 exposure on days 21 and 28 (Figure 3a,e). The same was true for stimulation experiments with 2 µg/mL on every time point compared to the control. In addition to gene expression and protein quantification after BMP-2 exposure, mineralization was quantified (Figure S1). BMP-2 stimulation did not significantly increase mineralization after 7 and 14 days compared to the control. The only significant increase was visible for 2 µg/mL BMP-2 after 14 days compared to 7 days (*p* = 0.0092).

### 2.3. Morphological Changes of Human Pre-Osteoblasts Due to Long-Term BMP-2 Stimulation

**Figure 4:**
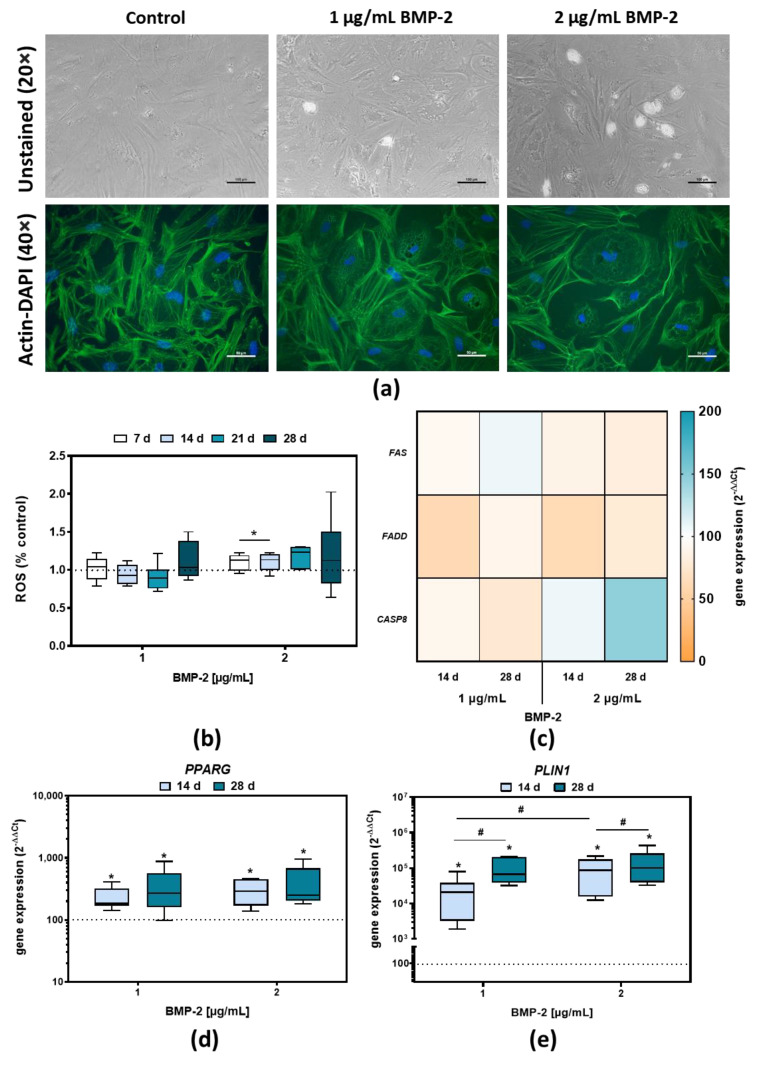
Long-term effect of BMP-2 exposure in human pre-osteoblasts. (**a**) Unstained microscopic images (bar: 100 µm) and actin-DAPI-stained images (bar: 50 µm) after BMP-2 exposure for 28 days with 1 or 2 µg/mL; (**b**) Reactive oxygen species (ROS) quantification after 7-, 14-, 21-, or 28-days following BMP-2 treatment with 1 or 2 µg/mL. The data are depicted relative to the control values and are shown as medians, interquartile ranges, minimum, and maximum (n ≥ 6). (**c**) Gene expression analyses of *FAS*, *FADD*, and *CASP8* following BMP-2 exposure with 1 or 2 µg/mL after 14 or 28 days. The data are depicted as 2^(−∆∆Ct)^ values (n ≥ 7). (**d**,**e**) Gene expression analyses of *PPARG* and *PLIN1* mRNA transcripts following BMP-2 treatment with 1 or 2 µg/mL after 14 or 28 days. The data are depicted as 2^(−∆∆Ct)^ values (n ≥ 7). Significance was analyzed by two-way ANOVA with Bonferroni’s multiple comparisons test with * *p* < 0.05 compared to the control and ^#^ *p* < 0.05 compared to different time points or concentrations.

In our cell experiments, it was observed that either after 14 days or especially after 28 days, BMP-2 stimulated pre-osteoblasts exhibited cellular inclusions (Figure 4a). Phalloidin-DAPI stain revealed a rearrangement of the actin cytoskeleton in the affected cells. The cytoskeleton was displaced by the inclusions and the actin crosslinks were no longer as prominent as in the unstimulated control (Figure 4a). To exclude cellular stress as the cause of these inclusions, the presence of reactive oxygen species (ROS) was quantified. In contrast to stimulation with 1 µg/mL BMP-2, a non-significant increase in extracellular ROS over the entire period was evident for the higher concentration. (Figure 4b). Additionally, the analysis of *FAS*, *FADD*, and *CASP8*, genes involved in apoptosis [30] did not show any significant influence of BMP-2 exposure on these processes (Figure 4c).

To investigate the inclusions in more detail, *PPARG* and perilipin 1 (*PLIN1)* were analyzed by gene expression. *PPARG* encodes a protein involved in adipogenic differentiation and *PLIN1*, a protein belonging to a family of lipid droplet surface proteins [31,32]. Both genes were significantly increased after BMP-2 exposure for 14 or 28 days (for all: *p* ≤ 0.0001) (Figure 4d,e). The increase in the gene expression level was particularly pronounced for *PLIN1*. In addition, the longer exposure time of 28 days led to a further increase in *PLIN1* expression (*p* < 0.0001) compared to BMP-2 exposure for 14 days.

**Figure 5:**
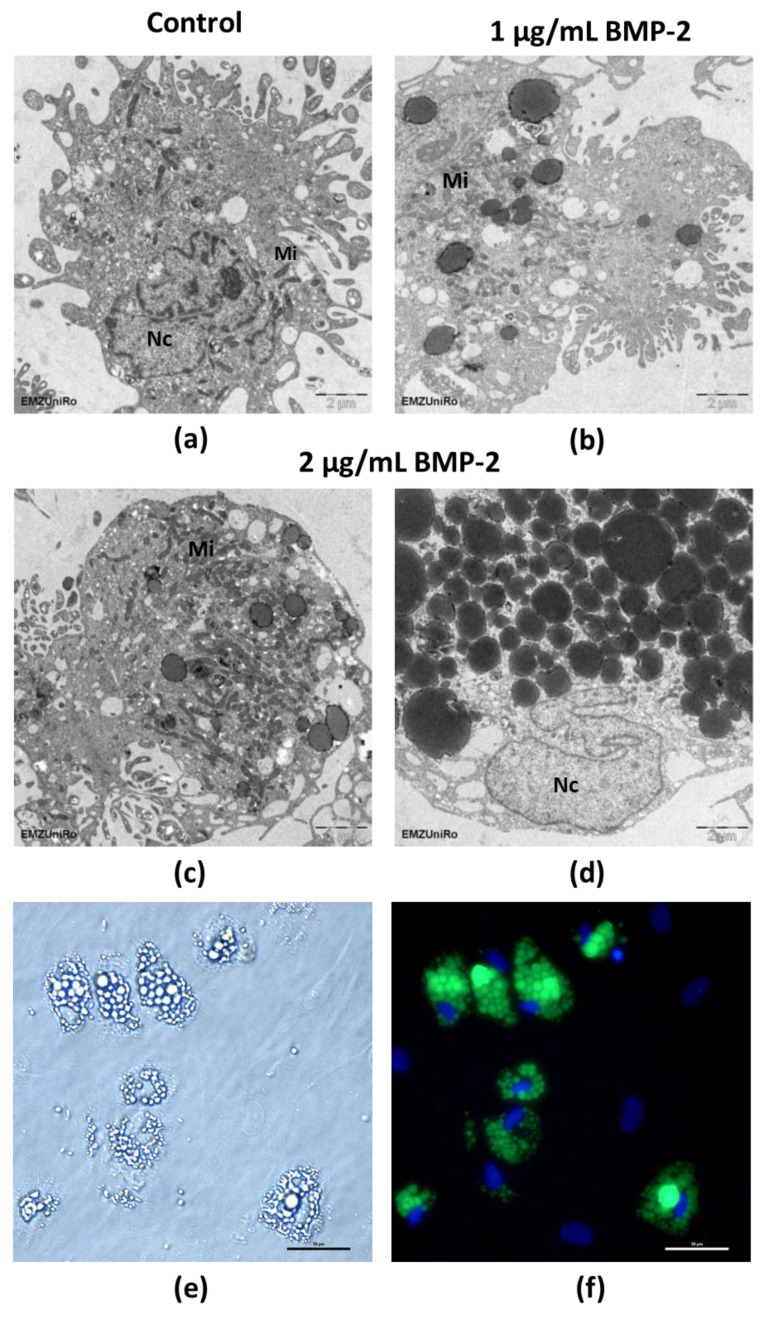
Cell morphological characterization of pre-osteoblasts stimulated with 1 and 2 µg/mL BMP-2 for up to 28 days. (**a**–**d**): Transmission electron microscopy images. After BMP-2 exposure, numerous osmiophilic inclusions were observed. After exposure to 2 µg/mL for 28 days, some cells contained a particularly large number of these inclusions; Nc: nucleus, Mi: mitochondria (bar: 2 µm); (**e**): Unstained microscopic image with intracellular inclusions following exposure to 2 µg/mL BMP-2 after 21 days (bar: 50 µm); (**f**): Bodipy staining of lipid droplets following exposure to 2 µg/mL BMP-2 after 21 days (bar: 50 µm).

By using transmission electron microscopy, detailed images of the intracellular inclusions were obtained (Figure 5a–d). It allowed detailed visualization of homogeneously filled, dark osmiophilic inclusions and empty vacuoles, appearing as bright spots (Figure 5b–d). Particularly high numbers of the dark osmiophilic inclusions were observed after exposure to 2 µg/mL BMP-2 over 28 days. Using scanning electron microscopy and energy-dispersive X-ray spectroscopy (EDS), no specific calcium signals could be detected in these inclusions, however, the analysis of the block-face area of the embedded pre-osteoblasts confirmed an accumulation of osmium (Figure S2). Osmium is used during post-fixation of the sample preparation for electron microscopy and is known for its strong interactions with (membrane) lipids, supporting the idea that these osmiophilic inclusions contain lipids. Furthermore, the pre-osteoblasts showing intracellular inclusions were stained with the green-fluorescent dye, Bodipy, to stain lipids and lipophilic compounds (Figure 5e,f). Thus, the inclusions appeared to be lipid droplets.

## 3. Discussion

Osteogenesis was promoted by 1 µg/mL and 2 µg/mL BMP-2 (in total 200 ng and 400 ng) for up to 28 days, as indicated by the increased ALP activity and enhanced gene expression levels of *ALPL*, *RUNX2*, *OSX*, *SPP1*, and *IBSP*, as well as the increased protein levels of ALP and OPN. Further, in a preliminary experiment, concentrations of 0.1, 1, and 10 µg/mL of BMP-2 (in total 20 ng, 200 ng, 2 µg BMP-2) were tested concerning osteogenic differentiation potential in pre-osteoblasts after 3 and 7 days of incubation. It was shown that there was no increased osteogenic differentiation potential with 0.1 µg/mL BMP-2 but with 1 and 10 µg/mL BMP-2. Between the higher concentrations, no significant differences could be observed (Figure S3). On the other hand, the gene expression, and protein quantification data on OPG and RANKL, after long-term exposure, suggest an increased osteoblastic potential of osteoclast activation after treatment with BMP-2. In addition, the appearance of cellular inclusions after 28 days of BMP-2 exposure was notable, suggesting possible adipogenic differentiation capacity.

### 3.1. Influence of BMP-2 on Osteogenic Differentiation Capacity

Increased osteogenic differentiation of BMP-2 stimulated pre-osteoblasts could be shown either on gene expression or protein level. The observed effects are not due to the osteogenic supplements (ascorbic acid, β-glycerophosphate, and dexamethasone), as shown in Figure S3. The addition of the osteogenic additives alone did not result in a significant increase of *RUNX2*, *OSX*, *ALPL*, and *IBSP*, compared to the cells cultivated without osteogenic additives. BMP-2 supplementation resulted in a significant increase of *OSX*, *ALPL*, and *IBSP* compared to the control, which was further enhanced by the addition of the osteogenic additives (Figure S4). RUNX2 acts as a master transcription factor, leading to the expression of osteoblastogenic markers, such as *ALPL*, *SPP1*, *IBSP*, *OSX*, and *COL1A1*, during osteoblastogenesis [4]. In our experiments, *RUNX2* expression was highest at 14 days and decreased at later time points, which is consistent with data from other studies, as RUNX2 decreases as maturation proceeds [33,34]. ALP was increased after BMP-2 exposure, indicating an initiated mineralization process of the bone cells [35]. However, the generated data did not show a *RUNX2*-mediated increased expression of *COL1A1*, which encodes the pro-alpha 1 chain of type I collagen, a highly relevant protein in the extracellular matrix formation in bone. Rather, a reduction in mRNA transcripts was observable. In bone, type I collagen comprises approximately 80% of the total proteins [36] and collagen fibrils serve to deposit Ca^2+^ and P_i_ to form hydroxyapatite crystals within their 40 nm holes [37]. The Ca^2+^ and P_i_ are presumably released from the extracellular matrix vesicles in which Ca^2+^ and P_i_ ions, enzymes, lipids, and miRNAs are enriched [38]. Furthermore, no change in *OCN* expression could be detected after BMP-2 exposure compared to the control, indicating an immature state in mineralization, since osteocalcin promotes the deposition of minerals in the extracellular matrix [5]. On the other hand, osteopontin, at the gene and protein level, and *IBSP*, at the gene level, were significantly increased by BMP-2 exposure, indicating an induction of matrix maturation [5,8]. OPN, especially p-OPN, regulates the hydroxyapatite deposition in the extracellular matrix [39]. Since this process is controlled by ALP, we assumed that a high ALP expression after BMP-2 exposure induces mineralization. Therefore, in further analysis, the mineralization via Ca^2+^ deposition was quantified, and no significant increase in mineralization through BMP-2 stimulation could be observed, and only a time-dependent increase in mineralization for 2 µg/mL BMP-2 stimulation was present (Figure S1). Since BMP-2 resulted in a lack of type I collagen, mineralization seems to be impaired after long-term exposure. This indicates that BMP-2 does not enhance final mineralization compared to unstimulated cells, due to the lack of collagen 1 deposition.

Moreover, BMP-2 exposure resulted in an increase in EGF protein compared to the control. EGF promotes osteoblast proliferation but leads to an inhibition of osteoblast differentiation in vitro [28,40,41]. Inhibition of EGF-mediated osteogenic differentiation is achieved by inhibiting the transcription factors RUNX2 and osterix [29]. However, since this effect could not be observed in our data, the exact mechanism of EGF, in this case, is unclear and needs further investigation. Though EGF inhibits the Wnt/β-catenin-induced osteoblast differentiation, it might be possible that osteogenic differentiation took place via BMP-2 and the SMAD pathway. Besides EGF, DKK1 is also a protein that acts as an inhibitor of the Wnt/β-catenin signaling pathway [42,43]. Because DKK1 was not affected by BMP-2 exposure compared to the control, it would be possible that DKK1 has no regulatory influence under these circumstances. Moreover, likely, EGF may already regulate this pathway.

### 3.2. Enhanced Inflammation and Osteoclastic Activation Potential Caused by BMP-2 Exposure in Human Pre-Osteoblasts

IL-6 and TNF-α are two pro-inflammatory cytokines, that can also induce osteoclastogenesis and subsequent bone resorption [44,45]. In our study, BMP-2 stimulation leads to a reduced secretion of IL-6, whereas TNF-α protein was enhanced. In the case of a highly inflammatory environment, both cytokines might be elevated compared to the unstimulated cells [46], indicating that no harshly inflammatory environment was present under BMP-2 exposure. On the other hand, TNF-α also plays an important role in bone healing, affecting mesenchymal stem cell (MSC) behavior in a dose- and time-dependent manner [47]. In an early inflammatory phase, TNF-α is released at low levels, thereby promoting bone repair [48], and in late phases of inflammation, higher doses are released, leading to bone destruction [47]. High TNF-α release leads to the inhibition of RUNX2 and OSX expression and thus to decreased levels of osteoblast-specific differentiation markers [49], which could not be observed in our data. This suggests that, although the amounts of TNF-α released were elevated compared to the control, no bone-destructive effect seems to occur.

The expression of OPN was increased on the gene- (*SPP1*) and protein levels. Although osteopontin is an important regulator protein in bone mineralization, it can also regulate osteoclastogenesis via binding to integrin αvβ3, which is of importance for the formation of a sealing zone in osteoclasts as well as for the activation of OPG expression in osteoblasts [50,51,52]. OPN, especially p-OPN, plays a major role in the control of hydroxyapatite deposition in the extracellular matrix [39], thereby inhibiting the mineralization of osteoblast cultures in a phosphate-dependent manner [52]. In further studies, the phosphorylation level of OPN after BMP-2 stimulation should be considered in more detail, as it regulates bone formation or resorption. Additionally, a reduction in OPG (gene and protein level) and the increase in RANKL after BMP-2 treatment compared to the unstimulated control were observed, indicating an enhanced osteoclast activation potential through the addition of BMP-2 to pre-osteoblasts. The RANKL-RANK signaling axis regulates the osteoclast differentiation and bone resorption, and OPG acts as a negative regulator in this signaling cascade by binding to RANKL and thereby hindering the interaction between RANKL and RANK [53,54,55,56]. Due to an 80-fold enhanced RANKL to OPG ratio (day 21, 2 µg/mL BMP-2) in this study, a clear impact on the induction of osteoclastic differentiation and bone remodeling processes can be derived.

### 3.3. Intracellular Inclusions in Human Pre-Osteoblasts after Long-Term BMP-2 Exposure

Following long-term BMP-2 stimulation of pre-osteoblasts, intracellular inclusions were detected that were presumably lipid droplets. Their dark, osmiophilic appearance could argue in this direction as well, however, their appearance is different from other lipid droplets. Therefore, Bodipy staining was performed, which confirmed that the intracellular inclusions contain lipids. Since lipid droplets are often associated with adipocytes [57], it is possible that adipogenesis was promoted by BMP-2. In agreement with these data, Seok et al. reported increased adipogenesis by rhBMP-2 in demineralized bovine bone particles and blocks grafted into the subperiosteal space of a rat calvarial bone [58]. Furthermore, there is the hypothesis that adipogenesis in bone marrow mesenchymal stem cells (BMSCs), the precursor cells of pre-osteoblasts, is the default pathway in the absence of osteogenic stimuli [59]. However, since BMP-2 is an osteogenic stimulus and a pro-osteogenic medium was used, this explanation seems unlikely. However, it could be a kind of transdifferentiation because BMSCs are the common progenitors for osteoblasts and adipocytes, which suggests the potential for transdifferentiation under certain physiological conditions [57,60]. The fact that *PPARG* and *PLIN1* are significantly increased in their gene expression in pre-osteoblasts after BMP-2 long-term exposure, compared to unstimulated cells, would fit this hypothesis.

Another possibility would be that the pre-osteoblasts store lipid droplets when exposed to pathological and aging conditions, as recently described for RUNX2-expressing pre-osteoblasts in models of Hdac3 conditional deletion and aging [61]. Therefore, the accumulation of reactive oxygen species (ROS) as an indicator of cellular stress was investigated, as well as the influence of BMP-2 long-term exposure on the expression of three apoptosis-associated genes (*FAS*, *FADD* and *CASP8*). However, no significant effects were detected in these analyses compared to the unstimulated control. BMP-2 seems to affect apoptosis in pre-osteoblasts, depending on the cell maturation state of the cells [23], but these effects occur soon after the BMP-2 addition and do not seem to be relevant after 14 or 28 days. Furthermore, lipid droplets also exist in non-adipogenic lineage cells, as the biochemical and metabolic pathways leading to lipid droplet formation and dissolution are not unique to adipocytes [57]. In osteoblast progenitors from bone marrow, the presence of lipid droplets is not as well-documented as in other cell types, however, there is recent evidence that BMSC-derived bone-formation cells may store intracellular fat when their differentiation is challenged—similar to skeletal muscle [57,62].

Overall, it must be taken into account that the cells used are primary human pre-osteoblasts that are present in a heterogeneous cell culture population. Despite the heterogeneous cell population, clear effects of BMP-2 regarding osteogenic differentiation, osteoclast activation potential via enhanced RANKL to OPG ratios, and adipogenesis could be observed. Nevertheless, in the case of osteoclastic effects, it must be determined in further studies to what extent the secreted proteins can promote osteoclastogenesis in monocytic precursor cells. In addition, since this study focuses on in vitro analyses only, the effects of BMP-2 long-term stimulation need to be validated in an in vivo study.

## 4. Materials and Methods

### 4.1. Isolation and Culture of Human Primary Pre-Osteoblasts

Human primary pre-osteoblasts (5 male donors, mean age: 63 ± 10 years; 12 female donors, mean age: 73 ± 12 years) were isolated from femoral heads of patients undergoing total hip replacement. All patients had signed a written agreement before the samples were collected. Before isolation, the Local Ethics Committee of the University of Rostock (registration no. A2010-0010) approved the study. For the isolation procedure, the previously described protocol was used [63].

Pre-osteoblasts were cultured in 25 cm^2^ culture flasks with osteogenic medium (Dulbecco’s modified Eagle’s medium (DMEM); PAN-Biotech, Aidenbach, Germany) supplemented with 10% fetal calf serum (FCS, PAN-Biotech, Aidenbach, Germany), 1% penicillin/streptomycin, 1% amphotericin B, 1% HEPES buffer, as well as the osteogenic additives 50 µg/mL of ascorbic acid, 1 mM β-glycerophosphate and 100 nM dexamethasone (all: Sigma-Aldrich, Munich, Germany) to maintain the osteoblastic phenotype. Incubation took place in a humidified atmosphere of 5% CO_2_ and at 37 °C. The medium was changed every second day. Reaching a confluence of 100%, the cells were transferred to a 75 cm^2^ culture flask, further cultivated up to a confluence of 100%, and then stored in 9:1 FCS:DMSO at −150 °C for subsequent use.

For the experiments, the cells were used in the third passage with an osteogenic cell culture medium containing the ingredients described above, while the amount of FCS was adjusted to 5%. A cell number of 1 × 10^4^ (in duplicates) was transferred into a well of a standard 48-well cell culture plate, allowing for cell adherence over 24 h at 37 °C and 5% CO_2_.

### 4.2. Exposure of Pre-Osteoblasts to Exogenous rh BMP-2

Recombinant human BMP-2 (100 µg) was dissolved in 100 µL of distilled water and further diluted in phosphate-buffered saline (PBS, Biochrom AG, Berlin, Germany) with 0.1% bovine serum albumin to obtain a concentration of 100 µg/mL of BMP-2. The preparation of the BMP-2 dilutions (1 and 2 µg/mL) was done with the osteogenic cell culture medium with additives. Human pre-osteoblasts were exposed to exogenous BMP-2 for up to 28 days.

### 4.3. Analysis of Metabolic Activity

The metabolic activity of human pre-osteoblasts after exposure to BMP-2 was determined by the metabolic activity assay water-soluble tetrazolium salt (WST-) 1 (Roche, Penzberg, Germany). The medium was removed, and cells were incubated with a defined volume of WST-1/medium reagent (ratio 1:10) at 37 °C and 5% CO_2_ for 45 min. Consecutively, supernatants were transferred into a 96-well cell culture plate (in duplicates) to determine the absorption at 450 nm (reference wavelength: 630 nm) in a microplate reader (Tecan Trading AG, Maennedorf, Switzerland).

### 4.4. Quantification of Enzymatic ALP Activity

For the quantification of ALP activity, the cells were washed twice with Tris-buffered saline (TBS; 137 nM NaCl, 2.7 M KCl, 50 mM Tris) and were then lysed for 10 min at RT using lysis buffer with 1% Tween 20 (Sigma-Aldrich, Munich, Germany) and 1 mM phenylmethylsulfonyl fluoride (PMSF; AppliChem, Darmstadt, Germany). Then, the measuring buffer containing 1 mM p-nitrophenyl phosphate (AppliChem, Darmstadt, Germany), 100 mM 2-amino-2-methyl-1,3-propanediol, and 5 mM MgCl_2_ (both: Sigma-Aldrich, Munich, Germany) was added to the cell lysates for 1 h at 37 °C. The reaction was stopped with 2 M NaOH (Merck, Darmstadt, Germany). The absorbance measurement was performed at 405 nm in a microplate reader (Tecan Trading AG, Maennedorf, Switzerland).

### 4.5. Analysis of Gene Expression

For RNA isolation, the peqGold Total RNA Kit (VWR International GmbH, Hanover, Germany) was used according to the manufacturer’s protocol. The RNA was eluted with RNase-free water and the RNA concentration was measured with a microplate reader and the NanoQuant^TM^ Plate (both: Tecan Trading AG, Maennedorf, Switzerland). Next, RNA was transcribed into cDNA using the High-Capacity cDNA Reverse Transcription Kit (Applied Biosystems, Foster City, CA, USA) and the related manufacturer’s protocol. A total of 45 ng of RNA was transferred into PCR tubes and was filled up with RNase-free water to a volume of 10 µL. Of the master mix, 10 µL were added to the RNA, and the PCR was done with the following protocol: 10 min at 25 °C, 120 min at 37 °C and 15 s at 85 °C in a thermocycler (Analytik Jena, Jena, Germany). In the end, 20 µL of RNase-free water was added to the samples. Before semi-quantitative PCR, samples were stored at −20 °C. For the relative quantification of target cDNA levels, the innuMIX qPCR DSGreen Standard was used in combination with the Qtower 2.0 (both: Analytik Jena AG, Jena, Germany). All primers used are listed in Table 1.

For each gene, a master mix with the respective forward and reverse primer was prepared, each with 0.5 µL, plus 3 µL of distilled water and 5 µL of the innuMIX qPCR DSGreen. From the samples, 1 µL of template cDNA was pipetted onto the bottom of a 96-well PCR plate in duplicates and a volume of 9 µL of the master mix was added. RNase-free water served as a negative control. The PCR plate was sealed and placed in the qTOWER to run the qPCR: 2 min at 95 °C, 40 cycles of 5 s at 95 °C and 25 s at 60 °C. A cycle threshold (Ct) of 30 was set as the limit of interpretation. For the interpretation of the results, the ∆∆Ct-method was used. The relative expression of mRNA was compared to the housekeeping gene HPRT (∆Ct = Ct_target_ − Ct_HPRT_) and from that ∆∆Ct-values, the relative amount of target mRNA in relation to the unstimulated control was calculated with the formula: ∆∆Ct = ∆Ct_treated_ − ∆Ct_tcontrol_.

### 4.6. Quantification of Protein Secretion in Cell Culture Supernatants

The protein amount of cross-linked C-telopeptides of Type I collagen (CICP) was quantified in cell culture supernatants using a MicroVue Bone CICP EIA ELISA (Quidel, San Diego, CA, USA) according to the manufacturer’s instructions. Absorbance was measured at 405 nm (Tecan Trading AG, Maennedorf, Switzerland) and the sample concentrations were calculated using a standard curve.

For all other protein quantifications in the cell culture supernatants, a custom-designed LEGENDplex™ (BioLegend^®^, San Diego, CA, USA) was used. The assay was performed according to the manufacturer’s instructions. The number of standards, samples, beads, and assay buffer was adjusted to 15 µL. Instead of incubating the samples with the premixed beads for 2 h at room temperature, an overnight incubation on the shaker at 4 °C was performed. For the measurements with the BD FACSVerse™ cell analyzer (BD Bioscience, Heidelberg, Germany), 500 events per sample were captured, and the data were analyzed using the BioLegend^®^ software (BioLegend^®^, San Diego, CA, USA). All measured values were set in relation to the total protein concentration in the samples. The total protein concentrations were quantified by the Qubit Protein Assay Kit and Qubit 1.0 (both: Invitrogen, Waltham, MA, USA).

### 4.7. Quantification of Reactive Oxygen Species

For the detection of free-reactive oxygen species, the supernatants of the BMP-2 long-term exposure experiments were collected at each media change and stored for analysis. The OxiSelect™ In Vitro ROS/RNS Assay Kit (Cell Biolabs, Inc., San Diego, CA, USA) and the manufacturer’s instructions were used for the ROS quantification. For analysis, 50 µL of standard or sample was transferred into a black 96-well cell culture plate (Thermo Fisher Scientific Inc., Waltham, MA, USA) and incubated with a catalyst for 5 min to speed up the oxidative reaction. In the next step, dichloro-dihydro fluorescein (DCFH) was added for 30 min, protected from light. The fluorescence signals were measured with a microplate reader (Tecan Trading AG, Maennedorf, Switzerland) at an excitation of 480 nm and an emission of 530 nm. For the determination of ROS content, a standard curve was used.

### 4.8. Cytoskeleton Staining

After 28 days of long-term BMP-2 exposure, the cytoskeleton was stained to determine morphological changes. Cells were washed with PBS and fixed in 4% paraformaldehyde for 10 min at RT. Afterward, the cells were washed with PBS and incubated with 0.05% Triton X (Merck KGaA, Darmstadt, Germany) for 5 min at RT to permeabilize the cell membrane. After two washing steps with PBS, the pre-osteoblasts were incubated with 100 nM Acti-stain 488 phalloidin (Cytoskeleton, Denver, CO, USA) for 30 min at RT and protected from light. The cells were washed three times with PBS. The staining of the nuclei was done with diamidino-2-phenylindole dihydrochloride (DAPI, Merck KGaA, Darmstadt, Germany) for 5 min at RT. Images were captured with a fluorescence microscope (Nikon ECLIPSE TS100, Nikon GmbH, Düsseldorf, Germany) at a 400× magnification.

### 4.9. Lipid Droplet Staining

The intracellular inclusions were stained with the green-fluorescent dye 4,4 -difluoro-1,2,5,7,8-pentamethyl-4-bora-3a,4a-diaza-s-indacene (Bodipy), which stains lipids, membranes, and other lipophilic compounds. For staining, cells were washed twice with PBS, fixed for 10 min with 4% PFA, and incubated with 0.05% Triton X (Merck KGaA, Darmstadt, Germany) for 5 min at RT to permeabilize the cell membrane. Then, the cells were washed three times with PBS and 2.5 µg/mL Bodipy solution (in 150 mM NaCl, Thermo Fisher Scientific Inc., Waltham, MA, USA) was added for 20 min. Consecutively, cells were washed twice with PBS and nuclei staining was performed with diamidino-2-phenylindole dihydrochloride (DAPI, Merck KGaA, Darmstadt, Germany) for 5 min at RT. Images were captured with a fluorescence microscope (Nikon ECLIPSE TS100, Nikon GmbH, Duesseldorf, Germany) at a 400× magnification.

### 4.10. Imaging by Transmission Electron Microscopy and Scanning Electron Microscopy

The human primary pre-osteoblasts were seeded in duplicate in a 6-well plate (50,000 cells/well) and 1 or 2 µg/mL BMP-2 was added after 24 h of incubation. Every seven days, a change of media was performed and thus the BMP-2 was renewed. After 28 days of BMP-2 exposure, the cells were detached, centrifuged, and fixed for transmission electron microscopy (TEM) analysis. Afterward, fixed cell pellets were washed in 0.1 M sodium phosphate buffer (pH 7.3), enclosed in 3% low melting agarose (Fluka, Munich, Germany), and stained with 1% osmium tetroxide (Roth GmbH, Karlsruhe, Germany) for two hours. The samples were dehydrated through a graded acetone series followed by an embedding in Epon resin (Serva, Heidelberg, Germany). The infiltration with resin started with a 1:1 acetone to resin mixture overnight, which was followed by infiltration with pure resin for over four hours. The samples were transferred to rubber molds and cured in an oven at 60 °C for at least 48 h. The specimens were cut from the resin blocks using a Leica EM Trim 2 mill (Leica Microsystems, Würzburg, Germany). Ultrathin sections (approximately 70–90 nm) were cut with a Reichert S ultramicrotome (Reichert, Vienna, Austria) using a diamond knife (Diatome, Nidau, Switzerland). The ultrathin sections were mounted on copper grids and contrasted with uranyl acetate and lead citrate. The ultrastructure was examined with a Zeiss EM902 electron microscope at 80 kV (Carl Zeiss, Oberkochen, Germany). Digital images were obtained with a side-mounted 1 × 2 k FT-CCD camera (Proscan, Scheuring, Germany) using iTEM Camera Control Imaging Software (Olympus Soft Imaging Solutions, Münster, Germany).

For scanning electron microscopy, resin blocks were mounted on aluminum stubs with adhesive carbon tape (Plano, Wetzlar, Germany). After coating with a thin layer of carbon (approx. 5 nm, Safematic CCU-010, Zizers, Switzerland) the respective block surface was examined in a scanning electron microscope (Merlin VP compact, Zeiss, Ober-kochen, Germany) equipped with an energy dispersive X-ray spectrometer for elemental analysis (Bruker XFlash 6/30 with Quantax QX400 and Esprit 2.0 software, Bruker Nano, Berlin, Germany) to analyze the elemental distribution in the embedded cells.

### 4.11. Statistical Analysis

Human pre-osteoblasts from a minimum of six independent donors were used for the experiments. If not otherwise stated, data are expressed as box plots with median, interquartile ranges, and minimum and maximum values using GraphPad Prism 8.0 (GraphPad Software, San Diego, CA, USA), and were analyzed by two-way analysis of variance (ANOVA) followed by Bonferroni’s multiple comparisons test. The level of statistical significance was set as *p* < 0.05. For statistical calculations, GraphPad Prism, version 8.0 was used, and adjusted *p* values are displayed.

## 5. Conclusions

Long-term exposure of human pre-osteoblasts to BMP-2 had a positive effect on osteogenic differentiation towards mature osteoblasts, however, proteins that promote osteoclast activation were also increased. The morphological changes of the cells after BMP-2 long-term exposure were particularly apparent. Intracellular inclusions occurred, which were likely lipid vacuoles, suggesting transdifferentiation or adipogenesis of the pre-osteoblasts. Overall, long-term exposure of human pre-osteoblasts with BMP-2 in lower concentrations did not resolve the described side effects, such as adipogenesis and osteoclastogenesis.

## Figures and Tables

**Table 1:** Primer sequences for qPCR.

Primer	Sequences (5′-3′)
Alkaline Phosphatase, Biomineralization Associated (ALPL)	fwd: CATTGTGACCACCACGAGAG
	rev: CCATGATCACGTCAATGTCC
Bone Morphogenetic Protein 2 (BMP-2)	fwd: GCGTGAAAAGAGAGACTGCG
	rev: ACCATGGTCGACCTTTAGGAG
Bone Morphogenetic Protein Receptor Type 1A (BMPR1A)	fwd: AGCCACATCTTGGAGGAGTCG
	rev: ATGTTTCCTGTGTACTGTCACCTT
Bone Morphogenetic Protein Receptor Type 1B (BMPR1B)	fwd: TGCCTTGTTGATAAAGGTTCAGAC
	rev: TTCCTGCACTTCGCAAAAGC
Bone Morphogenetic Protein Receptor Type 2 (BMPR2)	fwd: GGGTAAGCTCTTGCCGTCTT
	rev: CTGATAGTGCCAACCTCGCT
Caspase 8 (CASP8)	fwd: TGTTTTCACAGGTTCTCCTCCTTT
	rev: GAGAATATAATCCGCTCCACCCTT
Collagen Type I Alpha 1 Chain (COL1A1)	fwd: ACGAAGACATCCCACCAATC
	rev: AGATCACGTCATCGCACAAC
Fas Associated Via Death Domain (FADD)	fwd: ACCGAGCTCAAGTTCCTATGC
	rev: AAATGCTGCACACAGGTCTTC
Fas Cell Surface Death Receptor (FAS)	fwd: TGACCCTTGCACCAAATGTGA
	rev: AGACAAAGCCACCCCAAGTT
Hypoxanthine Phosphoribosyl-transferase 1 (HPRT)	fwd: CCCTGGCGTCGTGATTAGTG
	rev: TCGAGCAAGACGTTCAGTCC
Integrin Binding Sialoprotein (IBSP)	fwd: ATTTTGGGAATGGCCTGTGC
	rev: GTCACTACTGCCCTGAACTGG
Osteocalcin (OCN)	fwd: TCAGCCAACTCGTCACAGTC
	rev: GGTGCAGCCTTTGTGTCC
Osteoprotegerin (OPG)	fwd: TGTGGAATAGATGTTACCCTGTGTG
	rev: ACACTAAGCCAGTTAGGCGT
Osterix (OSX)	fwd: TAGGACTGTAGGACCGGAGC
	rev: CCATAGTGAACTTCCTCCTCAAG
Perilipin 1 (PLIN1)	fwd: AAGGGAAGAAGTTGAAGCTTGAGG
	rev: CACGCCCTTCTCATAGGCAT
Peroxisome Proliferator Activated Receptor Gamma (PPARG)	fwd: AGTCAGCCTTTAACGAAATGACC
	rev: CACGGAGCTGATCCCAAAGT
RUNX Family Transcription Factor 2 (RUNX2)	fwd: CGCCTCACAAACAACCACAG
	rev: ACTGCTTGCAGCCTTAAATGAC
Secreted Phosphoprotein 1 (SPP1)	fwd: AACGCCGACCAAGGAAAACT
	rev: GCACAGGTGATGCCTAGGAG

## Supplementary Materials

The following supporting information can be downloaded at: https://www.mdpi.com/article/10.3390/ijms23063077/s1.
