# Supplementary material for: BMP-2 Long-Term Stimulation of Human Pre-Osteoblasts Induces Osteogenic Differentiation and Promotes Transdifferentiation and Bone Remodeling Processes
Source: Int J Mol Sci. 2022 Mar 12;23(6):3077. doi: 10.3390/ijms23063077 (PMC8949995; doi:10.3390/ijms23063077)
Supplement: Supplementary file 1 [file ijms-23-03077-s001.zip › ijms-1595638-supplementary.pdf]

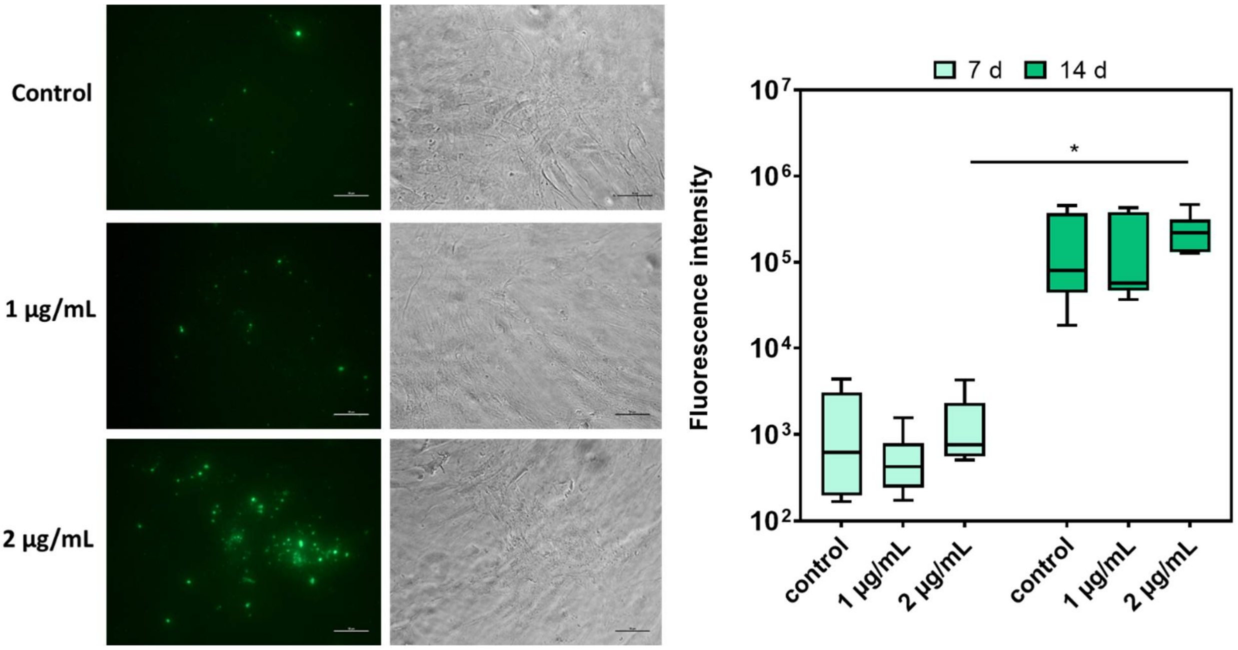

Figure S1: Quantification of mineralization with OsteoImage™ (Lonza, Walkersville, MD, USA) after 7 and 14 days of BMP-2 exposition (1 and 2 µg/mL, right) with fluorescence and light microscopic images after 14 days (bar: 50 µm) (left). The data are depicted as fluorescence intensities with medians, interquartile ranges, minimum, and maximum, * $p < 0.05$ compared to different concentration using two-way ANOVA with Bonferroni's multiple comparisons test ($n = 6$).

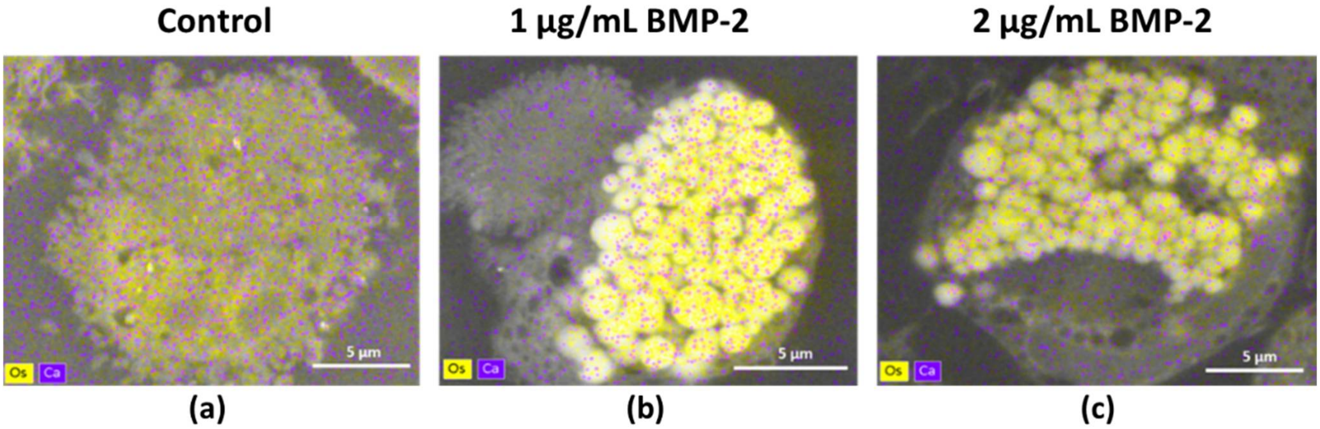

Figure S2. Elemental map for osmium and calcium using scanning electron microscopy and energy-dispersive X-ray spectroscopy (EDS) on the block-face area of the embedded pre-osteoblasts ((a) control, (b) 1 µg/mL, and (c) 2 µg/mL BMP-2) showing accumulation of osmium in cellular inclusions whereas specific enrichment of calcium cannot be detected. A calcium signal was lacking from the corresponding spectra of the samples, respectively (data not shown).

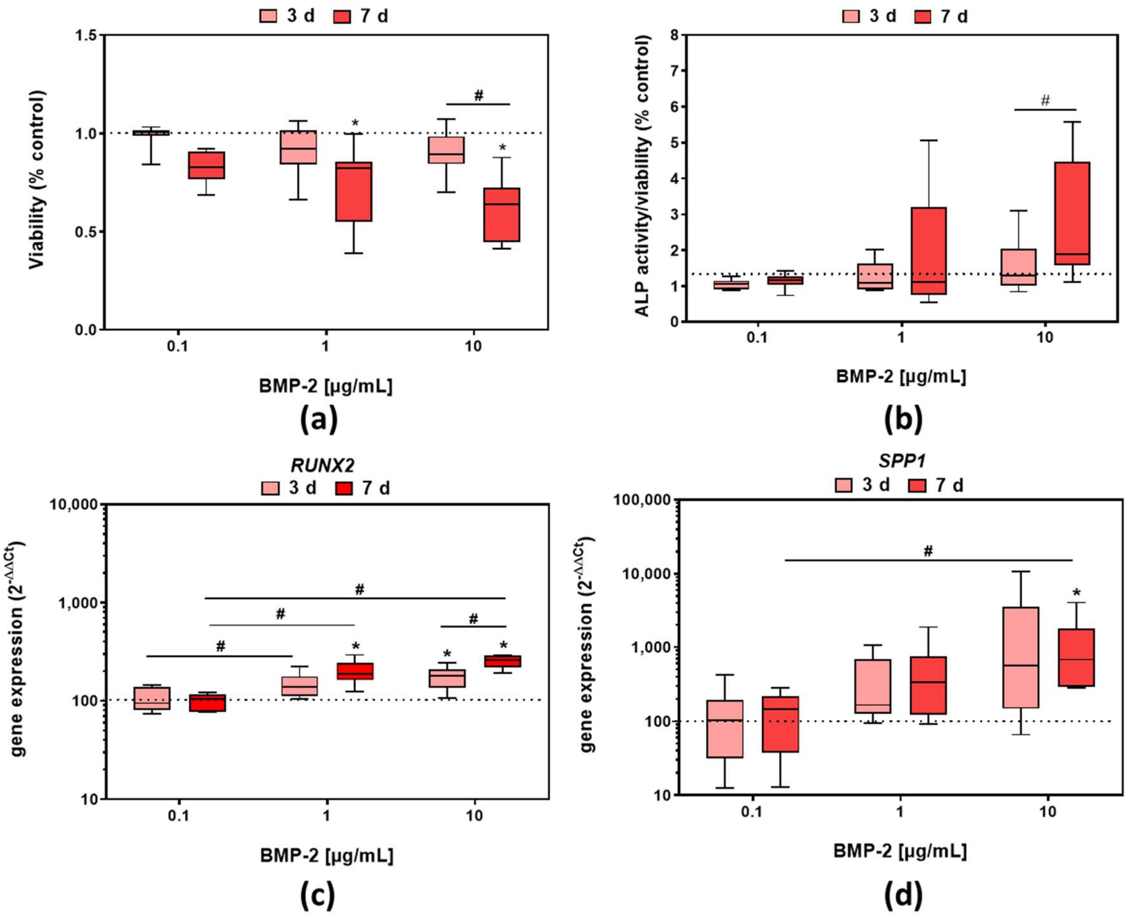

Figure S3. Viability (a), ALP activity related to viability (b), and gene expression of RUNX2 (c) and SPP-1 (d) of human pre-osteoblasts following BMP-2 exposure to 0.1, 1 or 10 µg/mL after 3 or 7 days. The data are depicted relative to the control values (a, b) or as $2^{(-\Delta\Delta Ct)}$ values (c, d). Results are depicted as medians, interquartile ranges, minimum and maximum with * $p < 0.05$ compared to control and $^{\#}$ $p < 0.05$ compared to different time points or concentration using two-way ANOVA with Bonferroni's multiple comparisons test ($n \geq 7$).

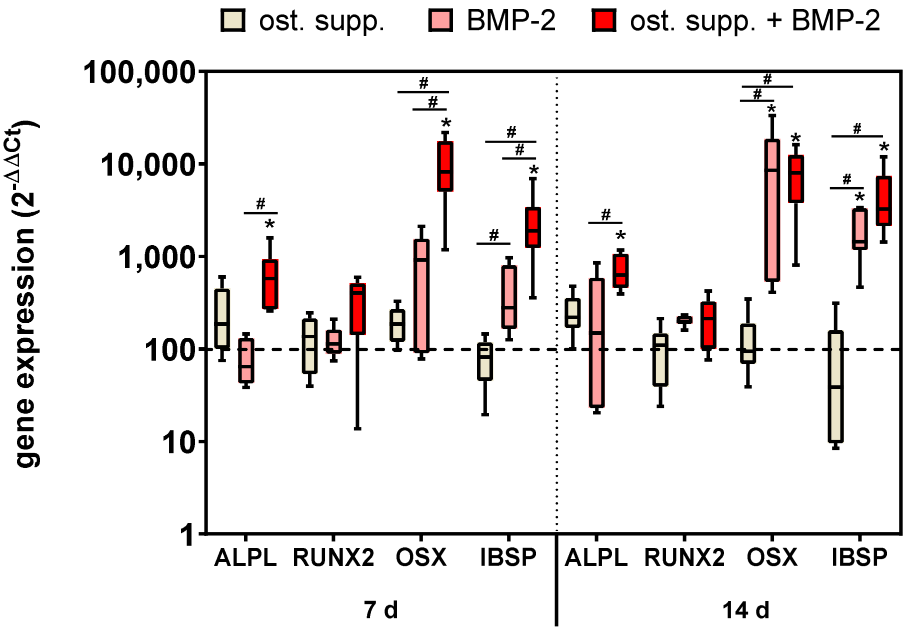

Figure S4: Gene expression of ALPL, RUNX2, OSX, and IBSP of human pre-osteoblasts following stimulation with osteogenic supplements (ost. supp.), BMP-2 (2 µg/mL) and a combination of both. The data are depicted as $2^{(-\Delta\Delta Ct)}$ values. Results are depicted as medians, interquartile ranges, minimum and maximum with * $p < 0.05$ compared to control and # $p < 0.05$ compared to different stimulation using two-way ANOVA with Bonferroni's multiple comparisons test (n = 6).
